# Optical power limiter in the femtosecond filamentation regime

**DOI:** 10.1038/s41598-021-93683-x

**Published:** 2021-07-12

**Authors:** Leonidas Agiotis, Michel Meunier

**Affiliations:** Department of Engineering Physics, Polytechnique Montreal, Montreal, QC H3C3A7 Canada

**Keywords:** Nonlinear optics, Optical techniques

## Abstract

We present the use of a power limiting apparatus to evaluate ultrafast optical nonlinearities of transparent liquids (water and ethanol) in the femtosecond filamentation regime. The setup has been previously employed for the same purpose, however, in a longer pulsewidth (> 20 ps) regime, which leads to an ambiguous evaluation of the critical power for self-focusing. The uncertainty originates from the existence of a threshold power for optical breakdown well below the critical power for self-focusing within this timeframe. Contrarily, using the proposed apparatus in the femtosecond regime, we observe for the first time a unique optical response, which features the underlying physics of laser filamentation. Importantly, we demonstrate a dependence of the optical transmission of the power limiter on its geometrical, imaging characteristics and the conditions under which a distinct demarcation for the critical power for self-focusing can be determined. The result is supported by numerical simulations, which indicate that the features of the observed power-dependent optical response of the power limiting setup are physically related to the spontaneous transformation of the laser pulses into nonlinear conical waves.

## Introduction

Prior to the z-scan first demonstration^1^, Soileau et al. introduced a passive optical power limiting device that relies on the self-focusing property of liquids^2^. The main idea of passive operation was based on the concept that a focused beam passing through a nonlinear medium will undergo strong phase change on its wavefront at increasing input powers, due to combined laser-induced breakdown and self-focusing inside the nonlinear medium. Thus, by placing an imaging lens after the nonlinear medium, one can observe limited transmission through a pinhole placed at the focus of that lens at high input powers.

In their original paper, the authors have employed their setup using nanosecond and picosecond pulses at an optical wavelength of 1.06 μm to study the nonlinear response of CS_2_. Indeed, the device has been tested to exhibit a “step-function”-like transmission for increasing input powers, of which the demarcation was identified as the critical power for self-focusing. The latter is generally defined as the required input peak power of the pulse above which self-focusing overcomes diffraction^3,4^. Effectively, the beam collapses so that its intensity increases and ionizes the medium. Nonetheless, for a pulse regime typically longer than 1 ps, optical breakdown is reached at a significantly lower power than the critical power for self-focusing within this timeframe due to the comparable times between energetic electron collisions and laser pulsewidth^3,5^. By contrast, for pulses in the femtosecond regime, self-focusing typically occurs rapidly, before optical breakdown is attained in the medium^5^. Hence, the use of the technique with laser pulses longer than 1 ps, can typically lead to an underestimation of the critical power for self-focusing.

In this work, we employ the foresaid optical power limiter in the femtosecond filamentation regime, and we measure ultrafast optical nonlinearities in deionized water and ethanol. In our proposed approach we introduce to the system a pinhole (~ 15 μm in diameter), much smaller than the imaged beam waist at this location (~ 32 μm in diameter), which ensures a significant variation on the recorded output transmittance, related to spatial transformation of the beam profile after the beam collapses into a filament. Thereby, we observe unique features on the optical response of the setup when the pinhole is placed at various positions with respect to the focal plane of the imaging lens and we demonstrate that the critical power for self-focusing, among with other nonlinearities, can be reliably evaluated by this technique in the femtosecond regime. Further, we discuss a comparison between experimental observations and numerical simulations related to the beam size in the far-field and its transformation into a nonlinear conical wave^6–8^ for the well-established case of water.

## Results and discussion

We follow a heuristic approach in the experimental procedure, by obtaining measurements for various locations of the pinhole with respect to the imaging plane of the system (“Methods”). The reasoning lies in the highly dynamic nature of laser filamentation^3^, which depends strongly on the beam propagation axis-z. Indeed, near the critical power, a nonlinear focus is initiated in the medium, moving backwards in z as the power increases and the beam collapses into a filament. Accordingly, the backwards nonlinear focus displacement inside the medium is expected to affect the position of the imaged beam waist in the far-field. In what follows, we present results collected at three different locations of the pinhole: (a) exactly at the imaging plane (the plane that corresponds to the formed focus after lens L2 ), i.e. at $z={z}_{i}$, (b) at 1.5 Rayleigh lengths ${z}_{R,i}$ before the imaging plane, i.e. at $z={z}_{i}-1.5\times {z}_{R,i}$ and (c) at 1.5 Rayleigh lengths after the imaging plane, i.e. at $z={z}_{i}+1.5\times {z}_{R,i}$. The factor $1.5$ is $\sim {\left|MA\right|}^{-1}$, where $MA$ denotes the effective magnification of the system. The latter is noticeably affected by the focal waist position inside the sample, due to linear refraction (“Methods/Experimental”). Effectively, cases (b) and (c) correspond to the limits of field of focus at the focal plane of lens L1 (“Methods/Theoretical”).

**Figure 1:**
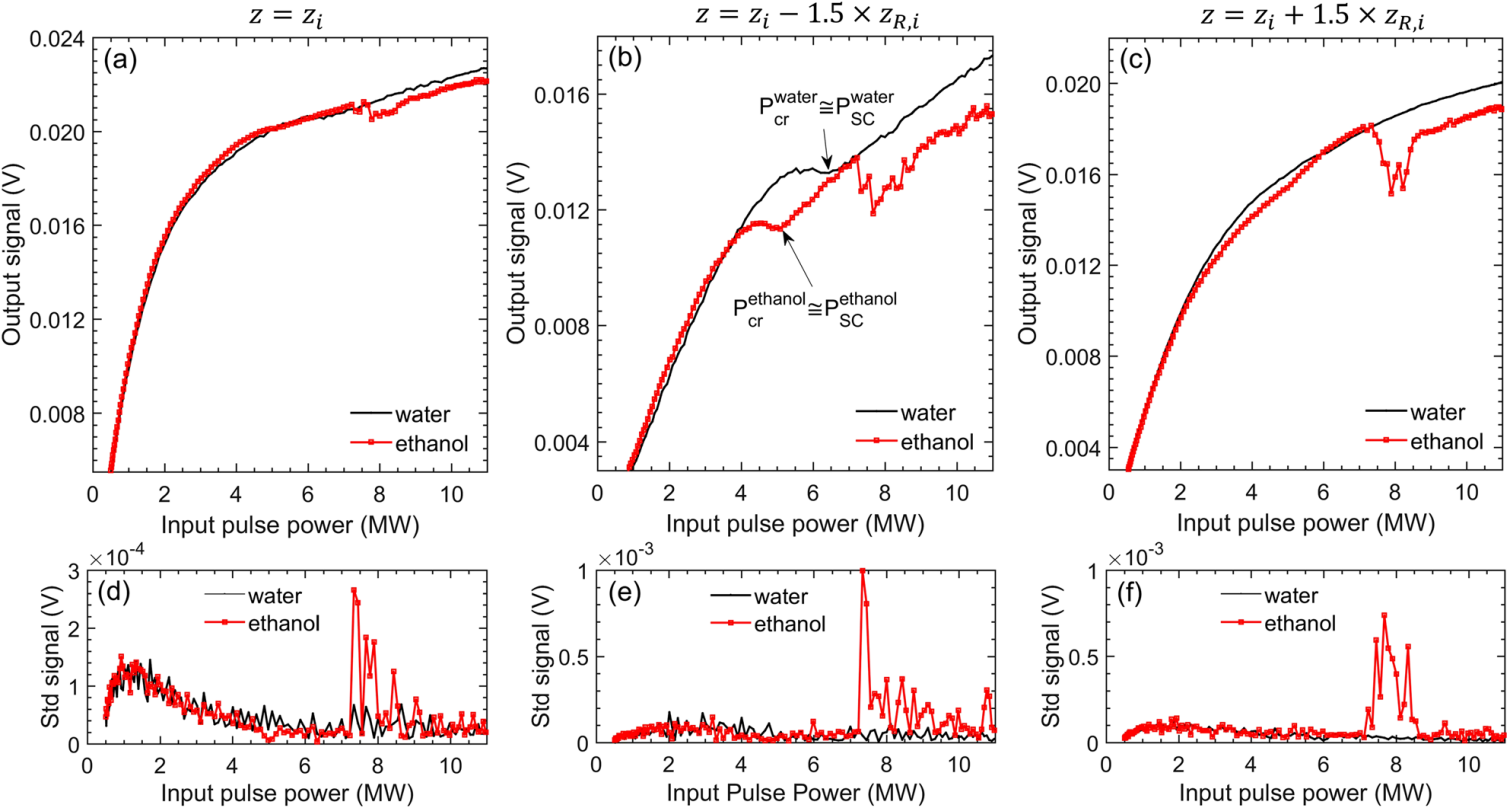
Typical experimental measurements in deionized water (black solid lines) and ethanol (red solid lines) by the optical power limiter in the filamentation regime. The top figures **(a–c)** present the output signal averaged over 10 shots and the bottom figures **(d–f)** show the standard deviation of these measurements. Top and bottom figures (presented in column pairs) correspond to different locations of the pinhole: **(a,d)** $\mathrm{z}={\mathrm{z}}_{\mathrm{i}}$, **(b,e)** $\mathrm{z}={\mathrm{z}}_{\mathrm{i}}-1.5\times {\mathrm{z}}_{\mathrm{R},\mathrm{i}}$ and **(c,f)** $\mathrm{z}={\mathrm{z}}_{\mathrm{i}}+1.5\times {\mathrm{z}}_{\mathrm{R},\mathrm{i}}$. Distinct features related to collapse of the beam become evident for case **(b)**. In all three cases, a decrease in optical transmission and sudden increase in the standard deviation of the measurement is observed for ethanol at an input power ~ 7.2 MW, which is identified as an optical breakdown threshold.

Figure 1 shows typical measurements in the examined liquids (water, ethanol). Case (a) is conceptually the same as the one of the original design of the device^2^, however, in the filamentation regime, it appears to bear different features. One cannot distinguish a “step-function”-like transmission, instead, a gradual, monotonic decrease of the latter is observed as the input optical power $P$ approaches the critical power for self-focusing ${P}_{cr}$. In addition, the response of the two liquids appears to be almost identical for both samples for $P<7.2MW\equiv {P}_{OB}^{eth}$, where ${P}_{OB}^{eth}$ stands for the optical breakdown threshold power for ethanol, which is discussed in the next section. Note that this threshold is evident in the signals of cases (b) and (c).

Contrarily to case (a), in case (b) features related to the collapse of the beam are observed. For both liquids, the optical transmission reaches a plateau (at around $\sim 5.3$ and $\sim\,4.2\,MW$ for water and ethanol respectively), which phenomenologically coincides with a strong phase modulation of the beam. The latter was apparent by visually inspecting the spatial profile of the beam when intercepted by a white card, as it acquires a redder color. After the foresaid plateau is reached, the optical transmission remains almost steady over a small $P$ interval. Above a critical input power, optical transmission increases monotonically again. It is that power that we identify as the critical power for self-focusing ${P}_{cr}$, since, as it will be shown later, it coincides with a beam width transformation and supercontinuum generation threshold ${P}_{SC}$ in water. The same conclusion can be generalized for the case of ethanol, owing to the similarity of the observed features on the power limiter response and of the qualitative observations on the beam width transformation into white light when intercepted by a white card for both liquids. Indeed, experiments have shown^9^ that the ${P}_{SC}$ is approximately equal to ${P}_{cr}$ with $\pm 10\%$ precision for a variety of optically transparent media, which is attributed to the universality of the physics of laser filamentation^5^. Importantly, our analysis, presented in the next paragraphs for the case of water, aims to demonstrate that ${P}_{cr}$ can be reliably determined by a standalone measurement by the power limiting method operated in case (b) geometry, presumably for a variety of transparent optical media. Finally, note that, despite the common signal features of both examined liquids in case (b), there is a clear difference in the value of $P$ that these features occur in each one of the samples, indicating the sensitivity of the measurement.

Case (c) exhibited a behaviour more like case (a), nonetheless, the transmitted signals exhibited a smaller slope as a function of $P$. In addition, ethanol measurement yielded a slightly weaker optical transmission compared to water at a $P$ interval before the total collapse of the beam into a filament, however, it was still difficult to identify the features discussed in case (b) for both samples.

Further, we note that the foresaid observed features are not affected significantly by losses related to nonlinear absorption at increasing input powers, which was confirmed in open-apertured optical transmission measurements through the cuvette when filled with the two liquids. Indeed, the optical transmission reduced only by ~ 1–2% at 6.8 MW for water and at 5.2 MW for ethanol, and by ~ 5% at 8 MW for water and at 6 MW for ethanol, respectively. Therefore, significant nonlinear losses occur only after the beam collapses into a filament, most likely due to increased plasma generation and direct multiphoton absorption in the liquids.

## Evaluation of nonlinearities

### Optical breakdown

The criterion for the determination of ${P}_{cr}$ as described by Soileau et al.^2^ in their original work, was established by monitoring the standard deviation of normalized transmission measurements through the pinhole (calculated over 5 shots at each input power). The authors observed that the value of the foresaid standard deviation increases by an order of magnitude at ${P}_{cr}$ and subsequently suddenly drops. A similar behaviour was observed in our experiments solely for the case of ethanol. The event was recorded only after an input pulse power of $\sim 7.2\,MW$, which was accompanied by a sudden drop on the optical transmission and a spark ignition inside the sample, as visually inspected to have been developed near the geometrical focus, implying the manifestation of optical breakdown.

As was demonstrated for example in^10^, typically electron densities of the order of 10^–18^ cm^−3^ are reached during filamentation by pulses of ~ 50 fs FWHM, via multiphoton ionization in a transparent medium, which is well below the critical plasma density of ~ 10^–21^ cm^−3^ for optical breakdown. The latter can be reached only by subsequent avalanche ionization, which depends implicitly on the focusing geometry (and explicitly on the developed intensity), on the optical properties of the medium, the ionization potential and the related cross section for cascade ionization^5,10^. Therefore, we attribute the observed spark ignition to favorable conditions for optical breakdown via avalanching in the case of ethanol at ~ 7.2 MW. Notably, simultaneous manifestation of both optical breakdown and filamentation is possible under certain focusing conditions inside a given sample, which is characterized by a decrease of the repetition rate of the pulse (here transformed in white light at the specified input power for ethanol), which was observed in our experiments and also reported in Fig. 6c of reference^10^.

### Critical power for self-focusing

**Figure 2:**
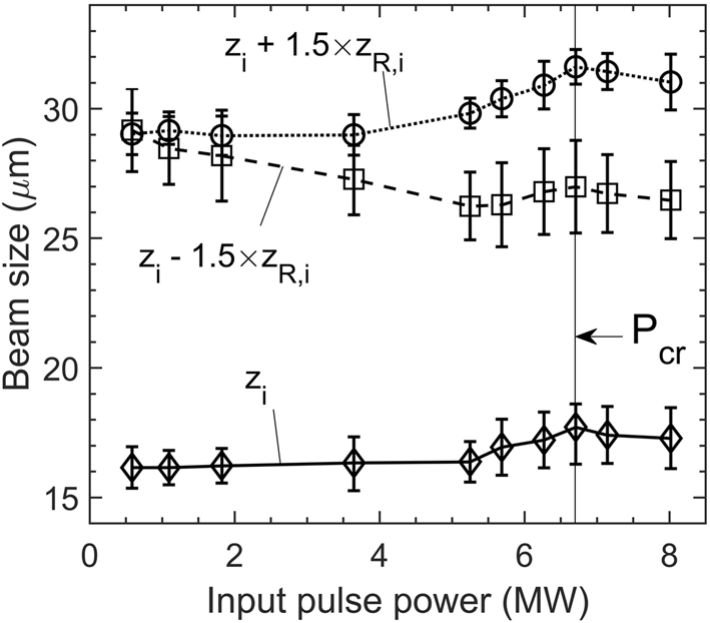
Imaged (far-field) beam size dependence on the input pulse power for deionized water at the three examined z-coordinates. In the far-field, a maximum divergence should correspond to a minimum beam size near the focal plane inside the sample. There is a strong implication of beam-width transformation due to the collapse, therefore, the local maximum at 6.7 MW is identified as the critical power for self-focusing ${\mathrm{P}}_{\mathrm{c}\mathrm{r}}$.

Further, we evaluated experimentally the power dependence of the far-field beam size (1/e^2^) near the imaging plane that is formed after L2 (see schematic in “Methods” section) when the cuvette is filled with water. The results (Fig. 2) show how the imaged beam size undergoes a transformation while $P$ approaches ${P}_{cr}$. Initially, it remains almost the same at ${z}_{i}$, it marginally increases at ${{z}_{i}+1.5\times z}_{R,i}$, while it reduces at ${z}_{i}-1.5\times {z}_{R,i}$ up to an input pulse of $\sim 5.3\,MW$, which coincides with the strong phase modulation observed experimentally. After that point, it increases for all three examined planes, presumably due to a shift in the angular divergence of the beam’s wavefront in the presence of strong self-phase modulation. The behaviour persists up to a critical power, (which we identify as ${P}_{cr}$) above which it drops for all planes. The initially opposing trend of beam size versus $P$ for $z={z}_{i}-1.5\times {z}_{R,i}$ compared to the rest two examined $z$, clarifies the signal behaviour in case (b) of Fig. 1. The beam size undergoes a sharper shift (reaching a local minimum) for $P\sim 5.3\,\mathrm{M}\mathrm{W}$, i.e., around the experimentally observed onset power for strong phase modulation of the pulse. Accordingly, such a sharp shift influences the recorded signal on the apertured detector since a larger beam size typically results in a decrease on the axial fluence of the beam. Finally, we note that the experimental 1/e^2^ beam size evaluation is approximate near ${P}_{c}$ since the beam is known to be gradually transformed into a nonlinear, Bessel-like conical wave upon collapse into a filament^7,8,11^.

### Supercontinuum generation

**Figure 3:**
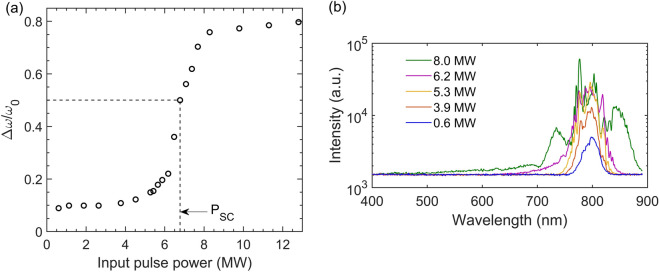
** (a)** Relative spectral broadening of the pulse as a function of the input power in deionized water. The estimation has been performed as in^12^. Note that the authors of^12^ have chosen $\mathrm{\Delta }\mathrm{\omega }/{\mathrm{\omega }}_{0}\left(\mathrm{P}={\mathrm{P}}_{\mathrm{S}\mathrm{C}}\right)\cong 0.5$, so here, ${\mathrm{P}}_{\mathrm{S}\mathrm{C}}$ is close to 6.7 MW. **(b)** Experimentally obtained supercontinuum spectra for water as a function of the input power.

Importantly, ${P}_{cr}$ seems to be very close to ${P}_{SC}$, defined as the dramatic increase on the pulse spectrum, an assessment that has also been drawn, for example, in references^9,10,12^. We illustrate this result by a measurement of the relative spectral broadening of the pulse as a function of the input pulse power in water (as performed in^12^). Based on the above analysis shown in Fig. 3, we conclude that ${P}_{cr}^{water}\cong {P}_{SC}^{water}\approx 6.7\,\,\mathrm{M}\mathrm{W}$ and we evaluate ${P}_{cr}^{ethanol}\cong {P}_{SC}^{ethanol}\approx 5.2\,\,\mathrm{M}\mathrm{W}$ from the optical limiter measurement [case (b) of Fig. 1].

### Nonlinear refractive index

Having marked the onset for ${P}_{cr}$ we derive the nonlinear index of refraction ${n}_{2}$ of the examined liquids from our measurements, accounting for the following: First, as it has been demonstrated by Fibich et al.^4^, we assume that the threshold for filamentation, must coincide with the critical power for self-focusing. The same author has demonstrated that for a Gaussian input spatial beam profile the critical power for self-focusing is inversely proportional to the nonlinear index of refraction, according to^4,13^

1 $${P}_{cr,0}=\frac{3.79{\lambda }^{2}}{8\pi {n}_{0}{n}_{2}}$$

Another factor considered, which significantly affects the critical power for self-focusing, is the beam propagation factor M^2^. We use a relation determined by Porras et al.^14^, which has been derived through a generalized ABCD propagation law and it reads

2 $${P}_{cr}={P}_{cr,0}{M}^{4}/\gamma$$

where $\gamma$ is a dimensionless factor related to the beam profile distribution and is equal to 1 for Gaussian profiles. Using Eqs. (1) and (2), ${M}^{2}=1.4$ (“Methods/Experimental”) and the values of the linear refractive index ${n}_{0}=1.33$ and 1.36 for water and ethanol respectively^15^, we have calculated ${n}_{2}$ for the examined liquids. The results are shown in Table 1 along with values cited in the literature, obtained by different techniques, for comparison.

**Table 1:** Evaluation of the Kerr nonlinear index of refraction of deionized water and ethanol: a comparison between values in the literature and our measurement.

Reference	Method	${n}_{2}\left({\times {10}^{-20}\mathrm{m}}^{2}/\mathrm{W}\right)$		Wavelength (nm)	Pulsewidth (FWHM)	Repetition rate
		Water	Ethanol			
^16^	Beam deflection^a^	$2.5\pm 20\%$	$3.2\pm 20\%$	800	150 fs	1 kHz
^17^	Ellipse rotation^b^	$3.4\pm 20\%$	$4.5\pm 20\%$	790	60 fs	NA
^21^	Spectral interferometry	$1.9\pm 10\%$		815	90 fs	1 kHz
^19^	Optical Kerr effect^c^	$1.9\pm \mathrm{N}\mathrm{A}$	$2.4\pm \mathrm{N}\mathrm{A}$	820	130 fs	76 MHz (chopped at NA frequency)
^10^	Supercontinuum onset	$2.0\pm \mathrm{N}\mathrm{A}$		810	45 fs	1 kHz
^22^	z-scan	$3.5\pm 1.9$		1150	90 fs	10 Hz
^18^	Optical Kerr effect^c^	$1.5\pm \mathrm{N}\mathrm{A}$	$2.9\pm \mathrm{N}\mathrm{A}$	1024	10 ps	150 MHz (100 shots)
Our work	Power limiting	$2.1\pm 20\%$	$2.7\pm 20\%$	800	55 fs	50 Hz

*NA* non available.

^a^Only the electronic ${\mathrm{n}}_{2}$ obtained in^16^ is considered.

^b^Larger pulsewidths are examined in^17^, however, only the smallest value is considered here, to exclude slower contributions on the value of ${\mathrm{n}}_{2}$.

^c^The value of ${n}_{2}$ in^18,19^ is given in ${10}^{-13}$ esu, thus we have calculated ${\mathrm{\chi }}_{1111}^{\left(3\right)}\left(\mathrm{e}\mathrm{s}\mathrm{u}\right)={\mathrm{n}}_{0}{\mathrm{n}}_{2}\left(\mathrm{e}\mathrm{s}\mathrm{u}\right)/\left(8\mathrm{\pi }\right)$ and converted in ${\mathrm{m}}^{2}/{\mathrm{W}}^{2}$ by applying the relation ${\mathrm{n}}_{2}\left({\mathrm{m}}^{2}/\mathrm{W}\right)=\left(3.95\times {10}^{-6}\right)/\left[{\mathrm{n}}_{0}^{2}{\mathrm{\chi }}_{1111}^{(3)}\left(\mathrm{e}\mathrm{s}\mathrm{u}\right)\right]$^20^.

In Table 1, the ultrafast (isotropic) nonlinear response of the two liquids, as evaluated by the power limiting method herein, is in fair agreement with measurements presented in the literature by various techniques at a wavelength around ~ 800 nm. For the case of water, at a longer wavelength of 1150 nm, the nonlinear refractive index is expected to increase as demonstrated experimentally in^22^, an observation that holds for increasing wavelengths up to 1250 nm. Further, we account that for data at a wavelength of 1024 nm^18^, a fair comparison of ${n}_{2}$ can still be performed with our measurements. In terms of pulsewidth excitation, Miguez et al.^17^ have demonstrated that for pulsewidth excitation shorter than 200 fs the ultrafast component of the nonlinearity remains almost unchanged for the two liquids. Although that according to that observation one would not expect a significant influence of the pulsewidth on ${n}_{2}$ value within the range of < 200 fs, the value of ${P}_{cr}$ might still be affected due to group velocity dispersion, while Eq. (1), which is a steady-state result^4^, is usually applied as a reference in the case of ultrafast pulses^3,4^. Further, results presented in^17^ imply that for pulses of 10 ps, ${n}_{2}$ can increase up to 40% (30% increase is discussed for ethanol in^16^) due to contributions from molecular reorientation. However this is not observed when comparing the values of ${n}_{2}$ reported by^18^ with the rest of the reported values shown in Table 1. The relative magnitude of the measured nonlinearities between water and ethanol is yet demonstrated. Finally, we should note that direct methods such as the z-scan technique, beam deflection, supercontinuum onset and the power limiting method typically require low repetition rate laser sources (< 1 kHz) to ensure that thermal effects are not affecting the evaluated nonlinearities.

## Theoretical interpretation

In what follows, we examine theoretically the experimental observations of the imaged (in the far-field) beam size dependency on $P$ in water. Let us first note that all three examined far-field beam profile distributions near the focal plane of imaging lens L2 are compressed by the same ratio ${d}_{i}/{d}_{o}\equiv \left|MA\right|$, where ${d}_{i}$ is the distance from L2 to the imaging far-field plane at ${z}_{i}$, ${d}_{o}$ is the distance of lens L2 from the focal plane of lens L1, located at ${z}_{f}$, and $MA$ denotes the linear magnification of the system. The latter remark imposes that, upon lens transformation, the planes near the focal plane of L1 at ${z}_{f}\pm {z}_{R,f}$ correspond to the imaging (far-field) planes at ${z}_{i}\mp {z}_{R,i}\times {\left|MA\right|}^{-1}$ (“Methods”).

**Figure 4:**
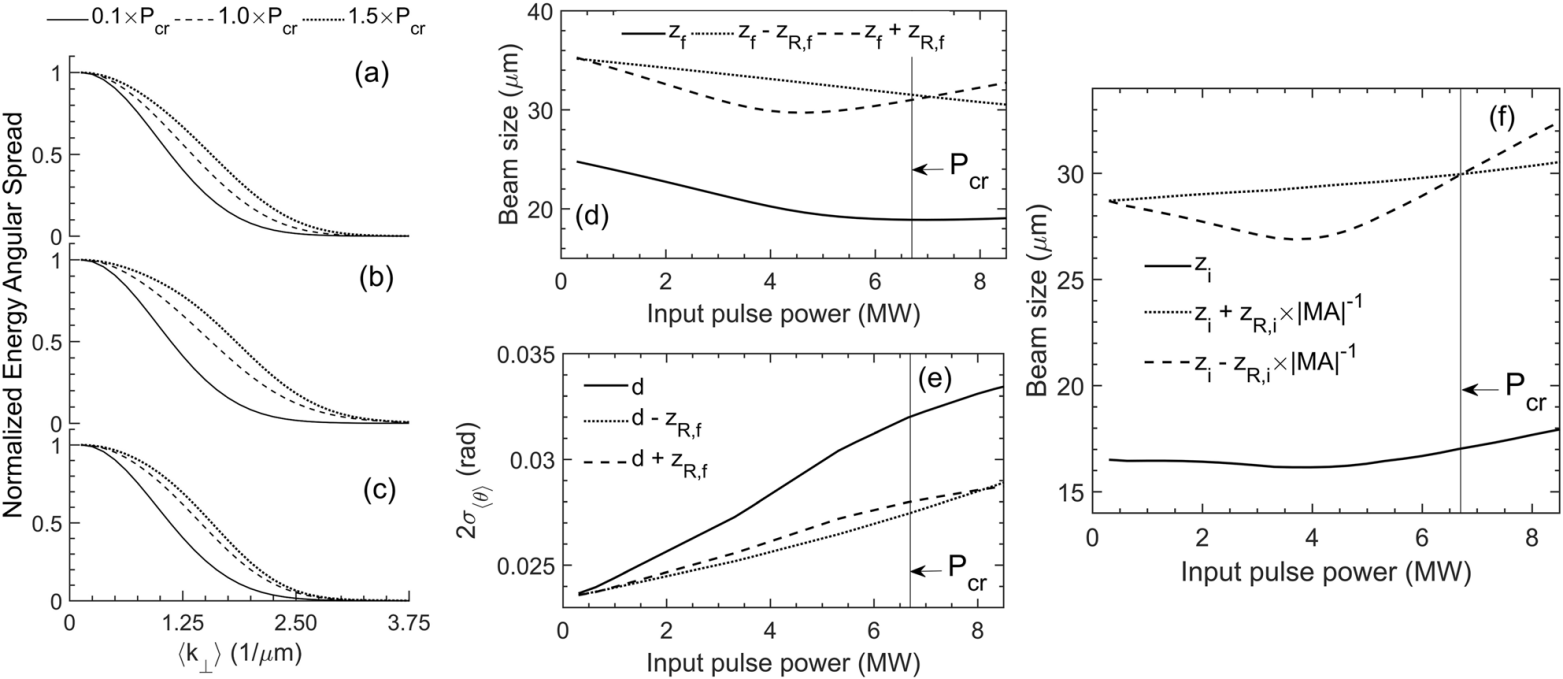
Numerical calculations based on Eqs. (3) and (4) (“Methods”) in water. **(a–c)** show the time-integrated and normalized far-field spectra $\mathrm{S}\left(\mathrm{t},{\mathrm{k}}_{\perp }\right)$ versus the time-averaged instantaneous transverse wavenumber $\langle{\mathrm{k}}_{\perp }\rangle$, calculated by the Hankel transformation of the solution of Eqs. (3) and (4) at **(a)** $\mathrm{z}={\mathrm{z}}_{\mathrm{f}}{-\mathrm{z}}_{\mathrm{R},\mathrm{f}}$, **(b)** $\mathrm{z}={\mathrm{z}}_{\mathrm{f}}$ and **(c)** $\mathrm{z}={{\mathrm{z}}_{\mathrm{f}}+\mathrm{z}}_{\mathrm{R},\mathrm{f}}$ respectively. The distributions are identified as the radiant energy angular spread, understood as the far-field counterpart of the pulse fluence (radiant energy exposure). **(d)** Beam size inside the propagation medium (near the focal plane of L1) versus input pulse power. **(e)** The standard deviation of $\langle{\mathrm{k}}_{\perp }\rangle$ taken from **(a–c)** has been used to calculate the standard deviation of divergence $\langle\mathrm{\theta }\rangle$ of the beam as a function of the input power at a distance $\mathrm{d}$ from reference ${\mathrm{z}=\mathrm{z}}_{\mathrm{f}}=0$ (first-order approximation). **(f)** Calculated imaged beam size in the far-field versus the input pulse power (first-order approximation).

The power dependence of the calculated beam waist size ${w}_{f}$ (at the focal plane of L1) inside the propagation medium (Fig. 4d) exhibits a behaviour different than the one measured at the imaging (far-field) planes, as expected. The imaged far-field beam size ${w}_{i}$ versus $P$ should be calculated instead and compared with the experiment. We first calculated the far-field electric field amplitude distribution $S\left(t,{k}_{\perp }\right)$ at an arbitrary distance $d\gg {w}_{f}$ from the examined z coordinates. We integrated in time (since the experimental measurements are time-integrated) to find the radiant energy angular spread distribution (shown in Fig. 4a–c) over a time-averaged instantaneous transverse wavenumber, i.e., $\langle{k}_{\perp }\rangle\equiv \frac{1}{T}\int_{0}^{T}dt{k}_{\perp }.$ From the resulting distributions we have calculated the second moments (twice the standard deviation ${\sigma }_{\langle{k}_{\perp }\rangle}$) of the power dependent $\langle{k}_{\perp }\rangle$. Assuming that the pulse undergoes only small spectral modulation before the critical power, we use the relation $\theta \approx {k}_{\perp }c/\left({\omega }_{0}{n}_{0}\right)$^11^, so that $2{\sigma }_{\langle\theta \rangle}\approx {2\sigma }_{\langle{k}_{\perp }\rangle}c/\left({\omega }_{0}{n}_{0}\right)$, to calculate the standard deviation of the divergence $\langle\theta \rangle$ at the fundamental frequency ${\omega }_{0}$ as a function of $P$. Apparently, this is an oversimplification when $P$ approaches ${P}_{cr}$, in view of strong dispersion while the spectrum of the pulse increases (Fig. 3). Thus, as shown in Fig. 4e, the divergence increases versus $P$, however, with expected deviations as $P$ approaches ${P}_{cr}$. Even so, as is, ${w}_{f}$ and $2{\sigma }_{\langle\theta \rangle}$ versus $P$ allow for a first order approximation of the imaged far-field beam size ${w}_{i}$ (see “Methods”), shown in Fig. 4f.

A good agreement is observed between experiment (Fig. 2) and simulations (Fig. 4f) up to $\sim 4.5\,\,\mathrm{M}\mathrm{W}$. The agreement gradually breaks down beyond that point, which is a result of the simplification $\langle\theta \rangle\approx \langle{k}_{\perp }\rangle \,\,c/\left({\omega }_{0}{n}_{0}\right)$. In fact, $\langle{k}_{\perp }\rangle$ can be related to the instantaneous frequency of the pulse in a self-phase modulation process, leading to the better approximation $\langle{k}_{\perp }\rangle\sim$ $\frac{{\omega }_{0}{n}_{0}}{c}\langle\theta \left(t\right)\left[1-{\frac{l}{c}\partial }_{t}n\left(t\right)\right]\rangle,$ where $n\left(t\right)$ and $\theta \left(t\right)$ are respectively the intensity (and implicitly time) dependent refractive index and divergence, and $l$ denotes the propagation distance of the pulse. Thus, it becomes evident that the former simplified relation between $\theta$ and ${k}_{\perp }$ does not hold as $P$ approaches ${P}_{cr}$ in view of the strong modulation of the pulse spectrum $\Delta \omega \propto \frac{l}{c}{\partial }_{t}n\left(t\right)$ (see Fig. 3). Dependency of phase modulation on $l$ also implies in practice that the observed signal features of the apparatus (Fig. 1) are dependent on the location of the focal plane inside the cuvette, which in turn is related to the effective magnification of the system due to linear refraction (see “Methods”).

In effect, upon collapse the beam transforms into a nonlinear conical (Bessel-like) wave and as a result the radiant energy angular spread varies at the generated wavelengths of the expanded spectrum surrounding the fundamental, according to the Fourier-space relation ${k}_{\perp }\left(\omega \right)=k\left(\omega \right)\mathrm{sin}\theta \left(\omega \right)$^3,6–8,11^. Consequently, the radiant energy angular spread of the fundamental wavelength will be limited upon this transformation beyond the critical point, as energy is transferred at other wavelengths and flows at high angles (X-wave formation for normal dispersion), forming a rim that surrounds the central spot of the generated white light.

## Conclusion

An optical power limiter has been employed to determine ultrafast optical nonlinearities of two transparent liquids (deionized water and ethanol) in the femtosecond filamentation regime. The technique has been utilized in the past only in longer pulse regimes (> 1 ps) leading to optical breakdown inside the samples, which typically occurs at optical pulse powers lower than the critical power for self-focusing. In contrast, in the femtosecond regime, the optical response of transparent liquids is governed by distinct features in the context of the studied technique. Particularly, we find that the threshold for self-focusing can be distinguished in the output signal when the apertured detection is located behind the conjugate focal plane of the imaging system, at a distance governed by the field of focus of the first focusing lens and the effective magnification. A presented theoretical analysis indicated how the effect is related to the spontaneous transformation of the beam into a nonlinear conical wave at the onset of filamentation.

Importantly, in this work, we demonstrated that the use of the power limiting setup in the femtosecond filamentation regime can be reliably utilized for future studies of ultrafast optical nonlinearities of various transparent materials, of which the evaluation is a challenging task even with the z-scan technique. Finally, the power limiting setup is anticipated to be a useful tool both for fundamental studies (e.g., competition between filamentation and optical breakdown) and the development of novel femtosecond laser filamentation based applications.

## Methods

### Experimental

**Figure 5:**
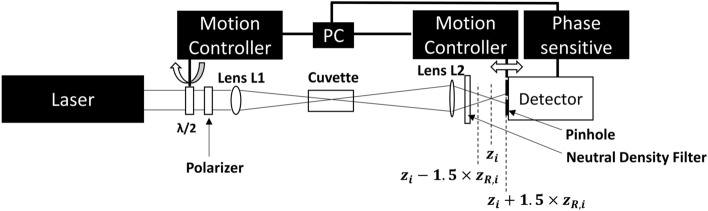
The optical power limiter experimental setup. A combination of a half-waveplate and a polarizer are used to control the power of the laser pulses. Two positive lenses are used to focus the beam on the sample and image onto the apertured (by a 15 μm pinhole) photodetector. The setup has been modified by placing a pinhole on a motor stage to allow translation towards z coordinate.

**Figure 6:**
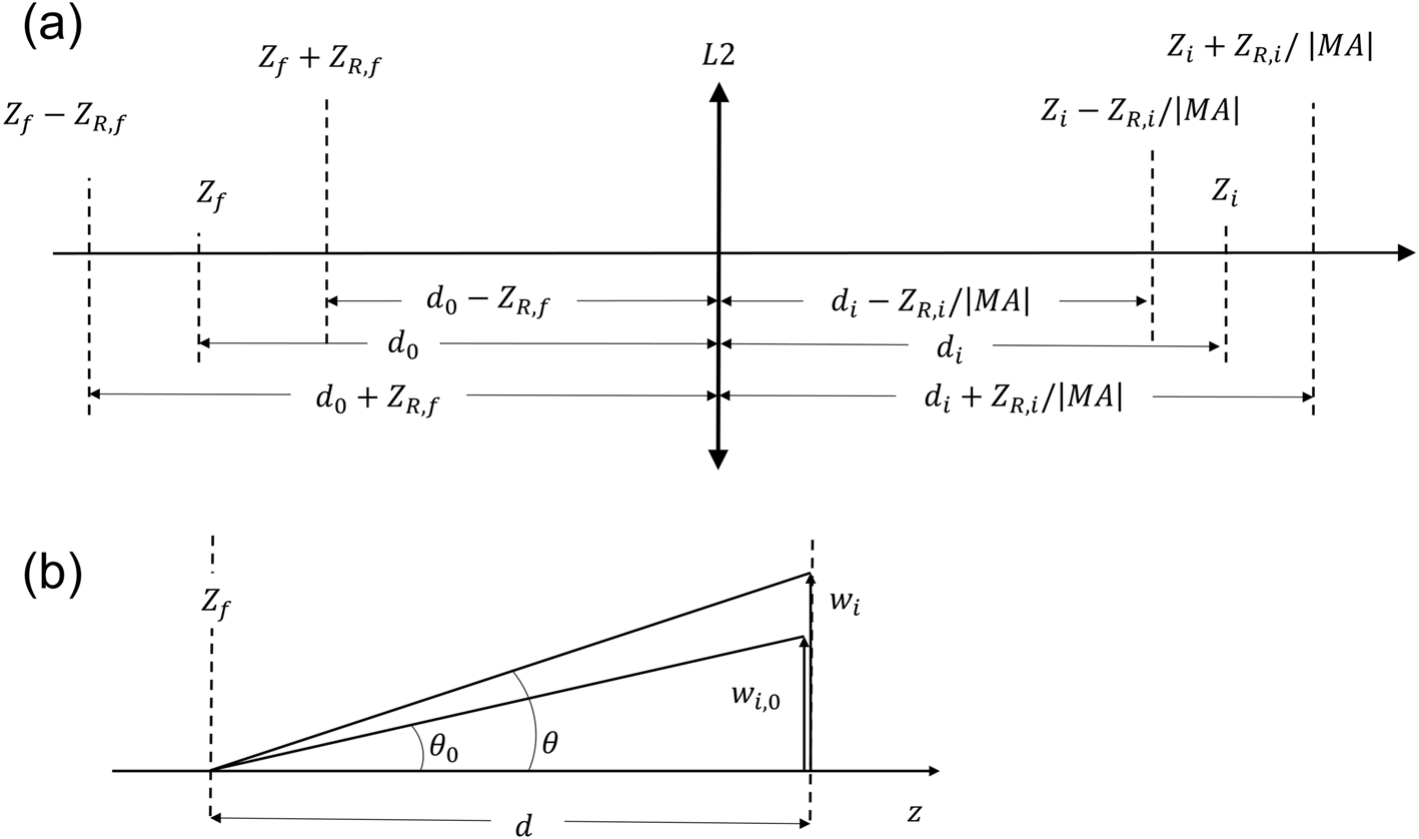
Conceptual diagrams used for calculation of the beam size at the imaging (far-field) planes. **(a)** Τhe correspondence of focal and imaging planes so that the linear magnification of the system MA remains invariant. **(b)** A diagram that shows how the beam size ${\mathrm{w}}_{\mathrm{i},0}$ at a distance d in the far-field shifts to ${\mathrm{w}}_{\mathrm{i}}$ when accounting for the power dependence of the divergence θ.

Our setup is shown in Fig. 5. We employed transform-limited pulses of 55 fs pulsewidth (defined by the full width at half maximum FWHM) produced by a Ti:Sapphire amplifier, operating at a repetition rate of 50 Hz. The laser beam, which had a Gaussian spatial profile and initial 1/e^2^ size of ~ 2.9 ± 1.5% mm, was collected by a lens L1 with a focal length of 200 mm. The beam waist of the focused beam in air was formed at approximately the same position with the focal plane of L1 and was estimated with a knife edge technique to be ${w}_{f}\sim 24.5\pm 3\%\,\mathrm{\mu }\mathrm{m}$ (1/e^2^), with a Rayleigh length ~ 1.680 mm. A second lens L2 with a focal length of 100 mm was positioned at a distance ${d}_{o}\sim 300\,\,\mathrm{m}\mathrm{m}$ apart from the focal plane of L1. Thus, L2 imaged the focused beam spot after ${d}_{i}\sim 150\,\,\mathrm{m}\mathrm{m}$ from its center with a magnification ${MA}_{0}\sim -0.5$ (Fig. 6a). Indeed, the focused beam waist after L2 was estimated to be ~ 12.4 ± 5% μm with a knife edge technique in air. The beam propagation factor M^2^ of the beam was measured to be ~ 1.4 after both L1 and L2 in air.

A 10 mm thick optical cuvette was positioned so as its center (5 mm from its entrance window along the beam propagation direction) coincided with the focal plane of L1. When filled with the examined liquids, the beam waist was estimated to be formed at $\sim {n}_{0}\times 5$ mm from the entrance window, due to the refractive index difference between the propagation mediums and air in the linear regime. Accordingly, the Rayleigh length at the focal plane of L1 is estimated ${z}_{R,f}\sim {n}_{0}\times 1.680\,\,\mathrm{m}\mathrm{m}$. These effects were confirmed experimentally by measuring the beam radius after the cuvette, when filled with water. Further, the addition of water in the optical cuvette, resulted in a change of the size of the beam collected by L2, a shift of the imaged spot position after L2, and also a change of the dimension of the imaged focal spot (${w}_{i}\sim 16.2\pm 5\%\,\,\mathrm{\mu }\mathrm{m}$) and Rayleigh length at the imaging plane (${z}_{R,i}\sim 0.745\,\,\mathrm{m}\mathrm{m}$), which were re-estimated by a knife edge technique in the linear regime. Accordingly, a shift of the effective linear magnification $MA\sim -0.67$ is observed (compared to ${MA}_{0}\sim -0.5$). Notably, ${w}_{i}$, ${z}_{R,i}$ and $\left|MA\right|$ increase when the focal plane is located closer to the output window of the cuvette in comparison to locations near the input window, because linear refraction affects the radius of curvature of the beam inside the examined medium^23^.

A pinhole (15 μm in diameter) was positioned approximately at the imaged focal plane formed after L2, when the cuvette was filled with water. The pinhole was placed at a motorized translation stage, which allowed fine adjustments over the beam propagation direction. The portion of beam that passes through the pinhole was collected by a photodetector. A neutral density filter was placed before the pinhole to attenuate the light intensity. The size of the pinhole allowed only a very low signal to pass through. On that account, the photodetector was connected to a lock-in amplifier for sensitive measurement of the generated output signal voltage. A variable attenuation of the beam was set by a combination of a half-waveplate and a polarizer. A motorised rotational stage allowed controlling of the waveplate by a personal computer, which was used to automate the measurements collected by the lock-in amplifier. Data were collected for each input power value and averaged over 10 laser shots. For each mean value of the data points, the standard deviation was calculated to estimate the statistical error. The measurement uncertainties presented in Table 1 are conservatively estimated to 20%, which include absolute uncertainties in $P$, due to laser energy fluctuations, pulsewidth, ${M}^{2}$ and γ value uncertainties, and relative uncertainties in the determination of ${P}_{cr}$.

For the measurements of the 1/e^2^ beam width in the far-field as a function of input pulse power, a knife edge technique was used. For the measurements of the relative spectral broadening $\Delta \omega /{\omega }_{0}$ presented in Fig. 3, we have replaced the pinhole and the detector shown in Fig. 5 with a fiber spectrometer and appropriate neutral density filters to collect the far-field spectrum of the pulse after propagation in water. We followed the methodology reported in^12^ so that $\Delta \omega ={\omega }_{b}-{\omega }_{r}$, where ${\omega }_{b}$ and ${\omega }_{r}$ stand for the maximum broadening towards bluer and redder frequencies respectively. The latter are recorded at the frequencies where the signal drops below the detection threshold, which we identify as 10% of the average baseline fluctuations (each measurement has been averaged over 10 shots). As reported in^9^, the selected signal level shall not change the result since the signal drops abruptly at the anti-Stokes wing. We further note that the apparatus cut-off on the red side (Stokes broadening) was at ~ 890 nm, possibly leading to an underestimation of the relative spectral broadening at high input powers (e.g. in Fig. 3, for $P>$ 8 MW). Nonetheless, we note that beyond that power, no significant further broadening occurs at the anti-Stokes wing for water. Besides, the idea of this approach was to quantify (with some uncertainty) the abstract definition of supercontinuum generation as the dramatic increase of the pulse spectrum when transmitted through the medium^12^.

### Theoretical

#### Numerical model

We used a theoretical model for the propagation of femtosecond laser pulses inside water (the most studied liquid medium of the above two)^24^. For a pulse propagating along the z axis, whose reference time frame moves at the group velocity ${u}_{g}$, the coupled system of differential equations that give the complex scalar envelope of the electric field $\widehat{\mathcal{E}}\left(r,\omega ,z\right)=\mathcal{F}\left\{\mathcal{E}\left(r,t,z\right)\right\}$ (written in Fourier space, where $\mathcal{F}\left\{\right\}$ stands for the Fourier transform, $r$ is the radial coordinate, $\omega$ is the radial carrier frequency, $t$ is the retarded time) and the electron density $\rho \left(r,t,z\right)$, reads

3 $$\frac{\partial \widehat{\mathcal{E}}}{\partial z}=\frac{i}{2\kappa \left(\omega \right)}{\Delta }_{\perp }\widehat{\mathcal{E}}+\frac{\left[{k}^{2}\left(\omega \right)-{\kappa }^{2}\left(\omega \right)\right]}{2\kappa \left(\omega \right)}\widehat{\mathcal{E}}+\frac{{k}_{0}}{\kappa \left(\omega \right)}\left[{iT}^{2}\left({\frac{{\omega }_{0}}{c}n}_{2}\right){\left|\widehat{\mathcal{E}}\right|}^{2}\widehat{\mathcal{E}}-T\frac{{\beta }_{K}}{2}{\left|\widehat{\mathcal{E}}\right|}^{2K-2}\widehat{\mathcal{E}}-\frac{i}{2{n}_{0}{\rho }_{c}}\left(\widehat{\rho \mathcal{E}}\right)\right]$$

4 $$\frac{\partial \rho }{\partial t}=\frac{{\beta }^{(K)}}{K\hslash {\omega }_{0}}\left(1-\frac{\rho }{{\rho }_{int}}\right){\left|\mathcal{E}\right|}^{2{\rm K}}$$

where ${\omega }_{0}$, ${k}_{0}$ is the pulse central frequency and central wavenumber respectively, $k\left(\omega \right)=\frac{\omega }{c}n\left(\omega \right)$, $n\left(\omega \right)$ is the linear refractive index of water^25^, ${\Delta }_{\perp }$ is the transverse Laplacian, $\kappa \left(\omega \right)={k}_{0}-\omega /{u}_{g}$, ${n}_{2}=2.1\times {10}^{-20}{\mathrm{m}}^{2}/\mathrm{W}$ is the experimentally evaluated nonlinear refractive index, ${\beta }^{({\rm K})}=1\times {10}^{-47}{\mathrm{c}\mathrm{m}}^{7}{\mathrm{W}}^{-4}$ is the multiphoton absorption cross section of water, $K=5$ is the required number of simultaneously absorbed photons of energy $\hslash {\omega }_{0}$ ($\hslash$ is the reduced Planck’s constant) to exceed the ionization potential of water ${U}_{i}=6.5\mathrm{e}\mathrm{V}$, $T=1+\frac{i}{\omega }\frac{\partial }{\partial t}$ is the self-steepening operator, ${\rho }_{c}={\varepsilon }_{0}{m}_{e}{\omega }_{0}^{2}/{e}^{2}$ is the critical plasma density (${\varepsilon }_{0}$ is vacuum’s dielectric permittivity, ${m}_{e}$ is the electron’s mass and $e$ is the elementary electric charge) and ${\rho }_{int}=6.68\times {10}^{22}{\mathrm{c}\mathrm{m}}^{-3}$ is the density of neutrals in the medium.

The initial conditions that are given to start propagation and match the experimental conditions, correspond to a Gaussian envelope distribution

$$\mathcal{E}\left(r,t,0\right)=\sqrt{\frac{2{P}_{in}}{\pi {w}_{0}^{2}}}{\mathrm{exp}}\left(-\frac{{r}^{2}}{{w}_{0}^{2}}-i\frac{{k}_{0}{r}^{2}}{2R}-\frac{{t}^{2}}{{t}_{p}^{2}}\right)$$

In this relation, ${w}_{0}=77\,\,\mathrm{\mu }\mathrm{m}$ (experimentally evaluated) denotes the input beam radius at the entrance of the cuvette, ${P}_{in}$ is the input peak power of the pulse, ${t}_{p}$ is the pulsewidth (related to the FWHM pulsewidth via ${t}_{FWHM}={t}_{p}\sqrt{2log\left(2\right)}$), $R=f-\frac{{z}_{R,f}^{2}}{f}$ is the radius of curvature, $f={n}_{0}\times 5\,\,\mathrm{m}\mathrm{m}$ is the axial distance that the beam waist is formed in the cuvette with respect to the entrance window and ${z}_{R,f}={n}_{0}\times 1.68\,\,\mathrm{m}\mathrm{m}$ is the experimentally evaluated Rayleigh distance, where ${n}_{0}$ is the refractive index of the medium at the central frequency (taken as 1 for air and 1.33 for water). To account for the effect of imperfect beam quality, we have multiplied the range of wavelengths $\lambda$ with the experimentally evaluated factor ${M}^{2}=1.4$, a transformation that can be used within the paraxial approximation to estimate time integrated quantities, where phase effects are of no consequence^26^.

#### Beam size at the focal and at the imaging (far-field) planes

We evaluated the input power dependence of the beam size at the examined z coordinates near the focal plane of L1 inside the propagation medium. To do so, we solved Eqs. (3) and (4) and we integrated the solutions in time, to determine the fluence of the pulse at a given z, and subsequently we calculated the beam size according to the second moments definition. Next, we evaluated the far-field distribution of the electric field amplitude $S\left(t,{k}_{\perp }\right)$ by performing a Hankel transform at the examined z-coordinates. The latter is a good approximation of the Fresnel–Kirchhoff integral, accounting for a plane of observation at a distance $d\gg {w}_{f}$, where ${w}_{f}$ denotes the beam waist at the focal plane of L1.

#### Observation planes near the focal and imaging planes

The observation planes at ${z}_{f}\pm {z}_{R,f}$ (around the focal plane of L1) are located ${d}_{0}\mp {z}_{R,f}$ away from L2, where ${d}_{o}$ is the distance from the focal plane of lens L1 to lens L2 itself (Fig. 6a). Therefore, since ${z}_{R,f}={z}_{R,i}/{\left(MA\right)}^{2}$, the corresponding imaging (far-field) planes should be located at a distance ${d}_{i}\mp {z}_{R,i}\times {\left|MA\right|}^{-1}$ apart from L2, so that the linear magnification of the system $MA=-{d}_{i}/{d}_{0}$ remains consistent. In other words, the observed electric field amplitude distributions at planes $z={z}_{i}\mp {z}_{R,i}\times {\left|MA\right|}^{-1}$ (i.e., at ${d}_{i}\mp {z}_{R,i}\times {\left|MA\right|}^{-1}$ away from L2) are equivalent to the ones calculated by the Hankel transform of the electric field amplitude distributions at planes $z={z}_{f}\pm {z}_{R,f}$ (i.e., at ${d}_{0}\mp {z}_{R,f}$ behind L2).

#### Imaged (far-field) beam size calculations

The power-dependent divergence $\theta$ over the divergence ${\theta }_{0}$ in the linear regime, quantifies the imaged far-field beam size change with respect to the linear regime, because $\frac{{w}_{i}}{{w}_{i,0}}\cong \frac{\theta }{{\theta }_{0}}$, where ${w}_{i,0}$ is the imaged far field beam size if $\theta ={\theta }_{0}$ (Fig. 6b). Considering the magnification of the optical system $MA$ in the linear regime, it holds ${w}_{i,0}=MA\times {w}_{f}$. Thus, the imaged spot size can be estimated as

5 $${w}_{i}\left({z}_{i}\right)\cong \left(MA\right)\times \frac{\theta }{{\theta }_{0}}{w}_{f}\left({z}_{f}\right).$$

Finally, we estimated the beam size at planes ${z}_{i}\pm {z}_{R,i}\times {\left|MA\right|}^{-1}$ starting from

$${w}_{i}\left({z}_{i}\pm {z}_{R,i}\times {\left|MA\right|}^{-1}\right)\cong {\left(MA\right)\times \frac{\theta }{{\theta }_{0}}}{w}_{f}\left({z}_{f}\pm {z}_{R,f}\times {\left|MA\right|}^{-1}\right)$$

Applying the paraxial equation $w\left(z\right)=\sqrt{1+{\left(z/{z}_{R}\right)}^{2}}$ for ${z}_{f}\pm {z}_{R,f}\times {\left|MA\right|}^{-1}$ and for ${z}_{f}\pm {z}_{R,f}$, we find:

6 $${w}_{i}\left({z}_{i}\pm {z}_{R,i}\times {\left|MA\right|}^{-1}\right)\cong {\left(MA\right)\times \frac{\theta }{{\theta }_{0}}\sqrt{\frac{1}{2}+\frac{1}{2{\left(MA\right)}^{2}}}}{w}_{f}\left({z}_{f}\pm {z}_{R,f}\right)$$

We used Eqs. (5) and (6) to plot Fig. 4f, where we have used data shown in Fig. 4e so that ${\sigma }_{\langle\theta \rangle}\to \theta .$
